# Oral Nystatin Prophylaxis for the Prevention of Fungal Colonization in Very Low Birth Weight Infants: A Systematic Review and Meta-Analysis of Randomized Controlled Trials

**DOI:** 10.7759/cureus.28345

**Published:** 2022-08-24

**Authors:** Abdulrahman Al-Matary, Lina Almahmoud, Raneem Masmoum, Sultan Alenezi, Salem Aldhafiri, Abdullah Almutairi, Hussain Alatram, Athbi Alenzi, Mohammed Alajm, Ali Artam Alajmi, Hadil Alkahmous, Fulwah A Alangari, Abdulrahman AlAnzi, Salihah Ghazwani, Ahmed Abu-Zaid

**Affiliations:** 1 Neonatology, King Fahad Medical City, Riyadh, SAU; 2 General Practice, Faculty of Medicine, The Hashemite University, Zarqa, JOR; 3 College of Medicine, Alfaisal University, Riyadh, SAU; 4 Pediatrics, Jahra Hospital, Al Jahra, KWT; 5 General Practice, Faculty of Medicine, Kuwait Institute for Medical Specializations, Kuwait City, KWT; 6 Emergency Department, Adan Hospital, Al-Ahmadi, KWT; 7 Pediatrics, Faculty of Medicine, Kuwait Institute for Medical Specializations, Kuwait City, KWT; 8 College of Medicine, Imam Mohammad Ibn Saud Islamic University, Riyadh, SAU; 9 Medicine, Jordan University of Science and Technology, Irbid, JOR; 10 Pediatrics, King Khalid University Hospital, Riyadh, SAU; 11 Internal Medicine, College of Graduate Health Sciences, The University of Tennessee Health Science Center, Memphis, USA

**Keywords:** meta-analysis, low birth weight, fungal colonization, fungal infection, nystatin

## Abstract

We conducted this systematic review and meta-analysis of randomized controlled trials (RCTs) to investigate the prophylactic role of oral nystatin in the prevention of fungal colonization in very low birth weight (VLBW) infants compared with placebo or no treatment intervention. From inception until June 2022, we screened four major databases for pertinent RCTs and examined their risk of bias. The main outcomes were the rate of fungal colonization, rate of invasive fungal infection, rate of mortality, mean length of stay in the neonatal intensive care unit (NICU), and mean duration of antibiotic treatment. We summarized data as risk ratio (RR) or mean difference (MD) with 95% confidence interval (CI), using the fixed-effects model. Five RCTs met our inclusion criteria. One RCT was evaluated as having "high risk," one RCT was evaluated as having "some concerns," and three RCTs were evaluated as having "low risk" of bias. Compared with the control group, oral nystatin prophylaxis was correlated with substantial decrease in the frequency of fungal colonization (n=4 RCTs, RR=0.34, 95% CI {0.24, 0.48}, p<0.0001), the rate of invasive fungal infection (n=4 RCTs, RR=0.15, 95% CI {0.12, 0.19}, p<0.0001), and the mean duration of antibiotic treatment (n=3 RCTs, MD=-2.79 days, 95% CI {-5.01, -0.56}, p=0.01). However, there was no significant difference between both groups regarding the rate of mortality (n=4 RCTs, RR=0.87, 95% CI {0.64, 1.18}, p=0.37) and mean length of stay in NICU (n=3 RCTs, MD=-2.85 days, 95% CI {-6.52, 0.82}, p=0.13). In conclusion, among VLBW infants, the prophylactic use of oral nystatin was correlated with favorable antifungal benefits compared with placebo or no treatment intervention.

## Introduction and background

The neonatal age group is at a higher risk of invasive fungal infection than other age groups, due to poor maturation of humoral and cell-mediated immunity [1]. Invasive fungal infection is a serious health problem that occurs in very low birth weight (VLBW) infants who are admitted to the neonatal intensive care unit (NICU) with a birth body weight of less than 1500 g. It composes 12% of all late-onset sepsis (LOS), of which 50% of the cases are caused by *Candida albicans* [2]. Fungal infections can be either nosocomial or vertical with a peak incidence between the second and third weeks of life [3]. Despite the medical intervention revolution, it is still associated with high mortality and morbidity rates [4].

Systemic or oral antifungal agents are commonly used as a preventive measure against candida colonization. They also decrease superficial fungal infection rates among high-risk patients [5]. Fluconazole is one of the commonly used prophylactic antifungals that is given either parenterally or orally and is systemically absorbed. Nystatin is another type of oral antifungal agent but it is not absorbable, thus allowing longer contact time with the colonizing fungi in the gastrointestinal tract [6,7]. Nystatin has several advantages, such as minimal side effects, lower cost than other antifungals, and easier to use [8]. However, previous systematic review and meta-analysis publications were of low evidence due to the paucity of reliable randomized controlled trials (RCTs) [8,9].

Consequently, we carried out this contemporary meta-analysis of RCTs to examine the benefits of oral nystatin versus control intervention (placebo or no treatment) in preventing the incidence of fungal colonization, invasive fungal infection, and mortality among VLBW infants. The proposed hypothesis is that prophylactic administration of nystatin will be associated with several beneficial antifungal outcomes compared with no prophylactic administration of nystatin among VLBW infants.

## Review

Materials and methods

The guidelines of the Preferred Reporting Items for Systematic Review and Meta-Analysis (PRISMA) [10] and the Cochrane Handbook for Systematic Reviews of Interventions were followed during the preparation of this research [11]. Owing to lack of direct involvement with human subjects, ethical approval was not necessary. This research was not retrospectively recorded in the International Prospective Register of Systematic Reviews (PROSPERO).

From inception until June 2022, we screened four major databases, namely Cochrane Central Register of Controlled Trials (CENTRAL), Scopus, Web of Science, and PubMed. The literature search strategy comprised preterm OR premature OR “low birth weight” OR LBW OR “very low birth weight” OR VLBW OR neonat* AND nystatin OR mycostatin OR nilstat OR nyamyc. There were no restrictions based on publication date or language.

The inclusion criteria comprised of the following: (1) patients - VLBW infants, (2) intervention - oral nystatin, (3) comparator - placebo or no treatment, (4) outcomes - rate of fungal colonization, rate of invasive fungal infection, rate of mortality, mean length of NICU stay, and mean duration of antibiotic treatment, and (5) study design - published RCTs. The exclusion criteria comprised all studies without RCT designs.

For study selection, duplicate citations were omitted and the remaining citations were subjected to screening of titles and abstracts and followed by full-text screening for final eligibility decision. Besides, the references of the included studies were further examined for possible missed RCTs. Two authors completed the study selection process and disagreements were resolved by discussion.

The Cochrane risk-of-bias assessment tool version 2 (The Cochrane Collaboration: London, UK) was used to examine the study quality and the overall judgments included "low risk," "high risk," or "some concerns" of bias [12]. Notably, publication bias was not explored owing to the small number of meta-analyzed studies [13].

For data extraction, two teams extracted the data; two authors per team independently completed the data extraction and disagreements were resolved by discussion. Details about the characteristics of the included studies (for example, country and study arms) and their patients (for example, gender and mean gestational age) were collected. Besides, we extracted data about select efficacy endpoints, such as rate of fungal colonization, rate of invasive fungal infection, rate of mortality, mean length of stay in NICU, and mean duration of antibiotic treatment.

The statistical analysis was performed using Review Manager (RevMan) software version 5.4 (The Cochrane Collaboration: London, UK) for Windows. Continuous data were pooled as mean difference (MD) with 95% confidence interval (CI), and the analysis was performed using the inverse-variance method. Also, dichotomous data were pooled as risk ratio (RR) with 95% CI, and the analysis was performed using the Mantel-Haenszel method. All analyses were completed using the fixed-effects model. Chi-square test with p<0.1 and I-squared (I^2^) statistic >50% established significant heterogeneity between studies [14]. Leave-one-out sensitivity analysis was performed to look into the stability of results after omitting one study at a time and then recalculating the effect size of the remaining studies. A p<0.05 declared statistical significance of the reported endpoints.

Results

**Figure 1:**
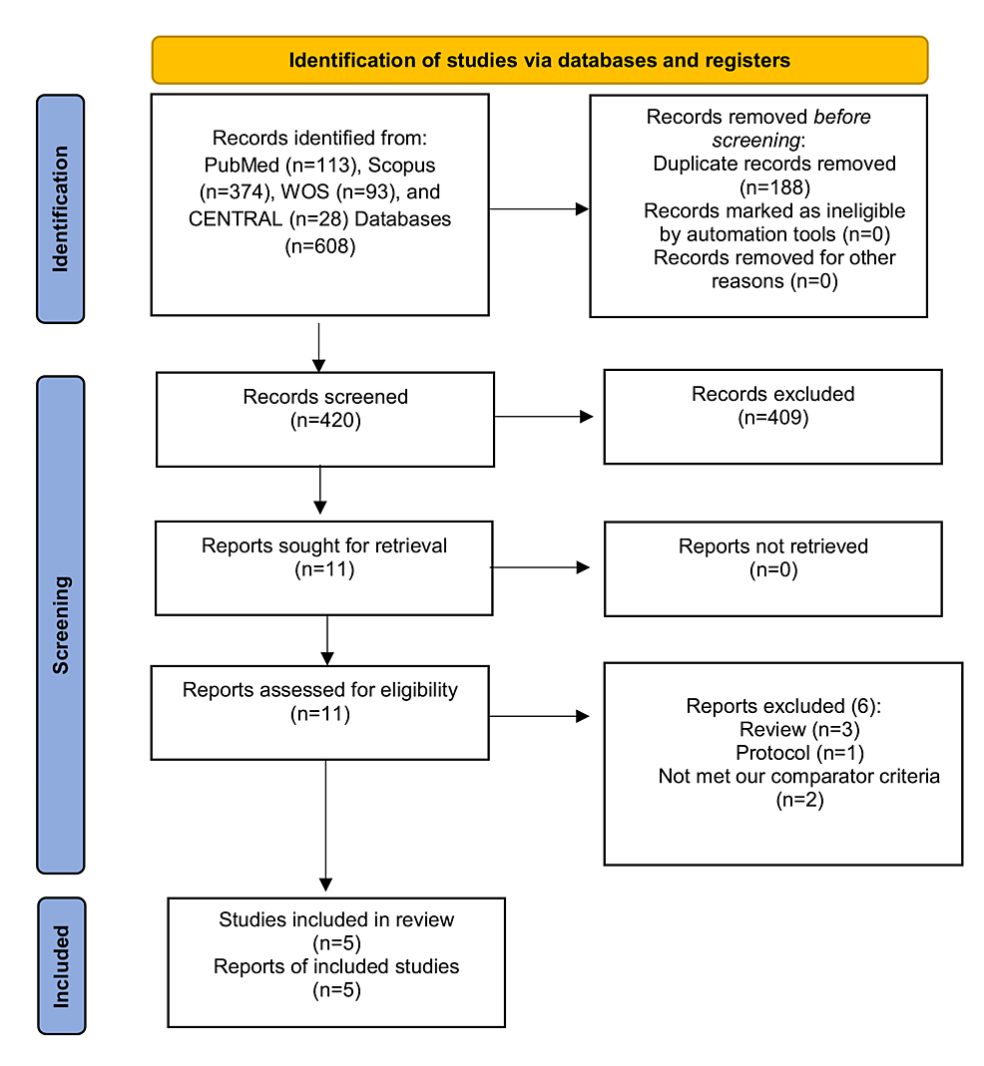
The Preferred Reporting Items for Systematic Reviews and Meta-Analyses (PRISMA) flow chart of literature search. CENTRAL: Cochrane Central Register of Controlled Trials

Figure 1 shows the PRISMA flow chart for our screening steps. Overall, five RCTs met our criteria and were included in the final analysis [7,15-18]. A total of 1750 participants were enrolled in these studies (n=884 participants were allocated to nystatin group and n=866 participants were allocated to the control group). The summary and baseline characteristics of the included studies are shown in Table 1 and Table 2, respectively.

**Table 1:** Summary of the included studies.

Study ID	Country	Trial duration	Total sample size, n	Study arms		Dose
				Intervention	Control	
Sims et al. (1988) [15]	United States of America	From June 1985 to May 1986	n=67	Nystatin (oral)	Nothing	Nystatin suspension (100,000 U/mL) every 8 hours
Ozturk et al. (2006) [7]	Turkey	From January 2002 to July 2005	n=1297	Nystatin (oral)	Nothing	Nystatin suspension (100,000 U/mL) every 8 hours
Aydemir et al. (2011) [16]	Turkey	From June 2008 to June 2009	n=185	Nystatin (oral)	Placebo	Nystatin suspension (100,000 U/mL) every 8 hours
Rundjan et al. (2020) [17]	Indonesia	From 2010 to 2012	n=95	Nystatin (oral)	Placebo	Nystatin suspension (100,000 U/mL) every 8 hours
Marzban et al. (2022) [18]	Iran	Not reported	n=106	Nystatin (oral)	Nothing	Nystatin suspension (100,000 U/mL) every 8 hours

**Table 2:** Baseline characteristics of the included studies. ELBW: extreme low birth weight; VLBW: very low birth weight

Study ID	Group	Sex, n (%)		Sample size, n	Gestational age (weeks), mean±standard deviation	Birth weight (g), mean±standard deviation	Vaginal delivery, (%)	Apgar score at 5 minutes, mean±standard deviation
		Male	Female					
Sims et al. (1988) [15]	Nystatin	Not reported	Not reported	33	27.2±1.72	898±195.31	Not reported	Not reported
	Control	Not reported	Not reported	34	27.4±2.33	918±239.07	Not reported	Not reported
Ozturk et al. (2006) [7]	Nystatin	Not reported	Not reported	657	Not reported	Not reported	Not reported	Not reported
	Control	Not reported	Not reported	640	Not reported	Not reported	Not reported	Not reported
Aydemir et al. (2011) [16]	Nystatin	50 (53.2)	44 (46.8)	94	28.7±2	1139±211	25	7±2
	Control	48 (52.7)	43 (47.3)	91	28±2.3	102±238	24	7±2
Rundjan et al. (2020) [17]	Nystatin	24 (51%)	24 (49)	47	30.8±2	1290±234.6	53.2	9±1.5
	Control	32 (66.6)	16 (33.4)	48	30.5±2.2	1318±259.2	62.5	8±1.75
Marzban et al. (2022) [18]	Nystatin	26 (49.1)	27 (50.9)	53	<28 months: n=9, 28-32 months: n=44	ELBW: n=12, VLBW: n=41	17	Not reported
	Control	27 (50.9)	26 (49.1)	53	<28 months: n=7, 28-32 months: n=46	ELBW: n=3, VLBW: n=50	20.80	Not reported

**Figure 2:**
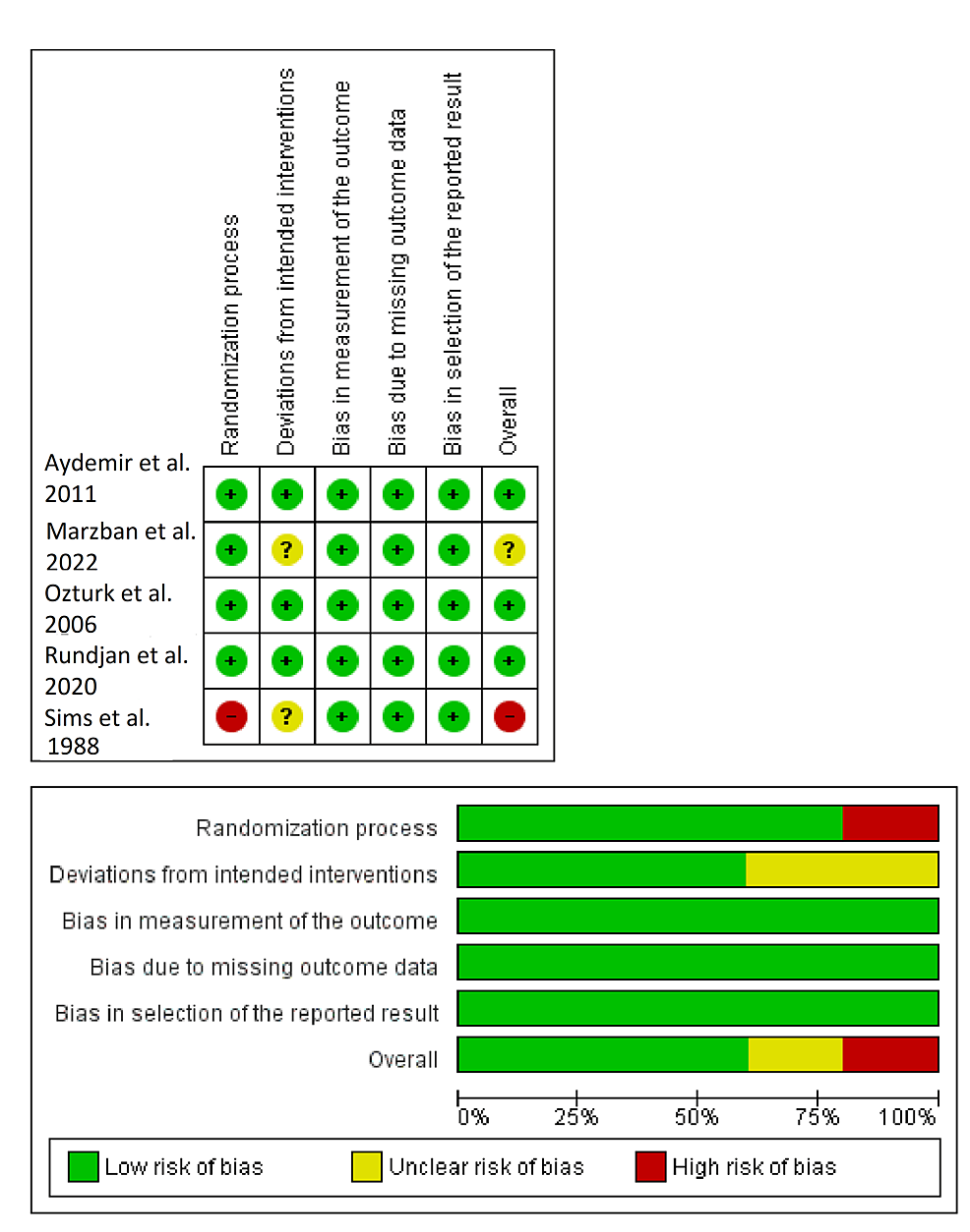
Summary of the risk of bias of the included randomized controlled trials.

Figure 2 shows the risk of bias summary of the included studies [7,15-18]. Three RCTs showed overall "low risk" of bias [7,16,17], one RCT revealed overall "some concerns/unclear risk" of bias [18], and one RCT revealed overall "high risk" of bias [14]. Sims et al. study was evaluated in the randomization process domain as "high risk" because the allocation sequence was not concealed [15]. Also, it was evaluated in the domain of deviations from intended interventions as "some concerns" because there was no information on whether there were deviations from usual practice that were likely to impact the outcome and were imbalanced between intervention groups. Similarly, Marzban et al. study evaluated deviations from intended interventions as "some concerns" because there was no information on whether there were deviations from usual practice that were likely to impact the outcome and were imbalanced between intervention groups [18].

**Figure 3:**
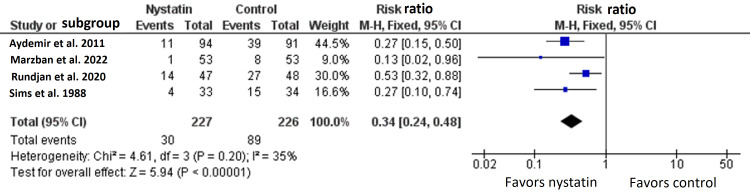
Meta-analysis of the rate of fungal colonization.

Four RCTs with 453 patients reported the rate of fungal colonization [15-18]. The overall effect size between nystatin arm and the control arm significantly favored nystatin arm (RR=0.34, 95% CI {0.24, 0.48}, p<0.0001) (Figure 3) [15-18].

**Figure 4:**
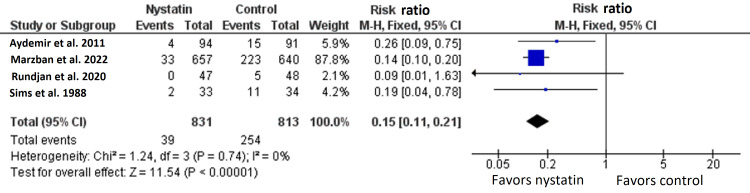
Meta-analysis of the rate of invasive fungal infection.

Four RCTs with 1644 patients reported the rate of invasive fungal infection [7,15-17]. The overall effect size between nystatin arm and the control arm significantly favored nystatin arm (RR=0.15, 95% CI {0.11, 0.21}, p<0.0001) (Figure 4) [7,15-17].

**Figure 5:**
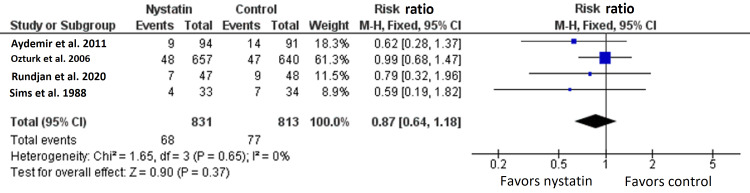
Meta-analysis of the rate of mortality.

Four RCTs with 1644 patients reported the rate of mortality [7,15-17]. The overall effect size between nystatin arm and the control arm did not significantly differ between both arms (RR=0.87, 95% CI {0.64, 1.18}, p=0.37) (Figure 5) [7,15-17].

**Figure 6:**
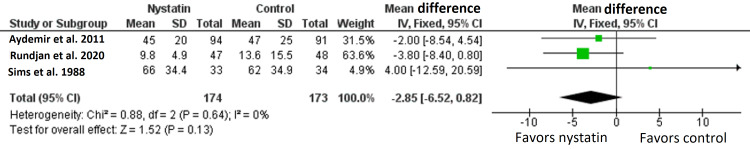
Meta-analysis of the mean length of stay in the neonatal intensive care unit.

Three RCTs with 347 patients reported the mean length of stay in NICU [15-17]. The overall effect size between nystatin arm and the control arm did not significantly differ between both arms (MD=-2.85 days, 95% CI {-6.52, 0.82}, p=0.13) (Figure 6) [15-17].

**Figure 7:**
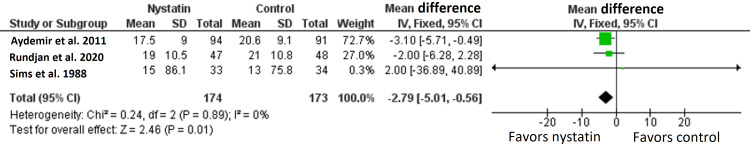
Meta-analysis of the mean duration of antibiotic treatment.

Three RCTs with 347 patients reported the mean duration of antibiotic treatment [15-17]. The overall effect size between nystatin arm and the control arm significantly favored nystatin arm (MD=-2.79 days, 95% CI {-5.01, -0.56}, p=0.01) (Figure 7) [15-17].

**Figure 8:**
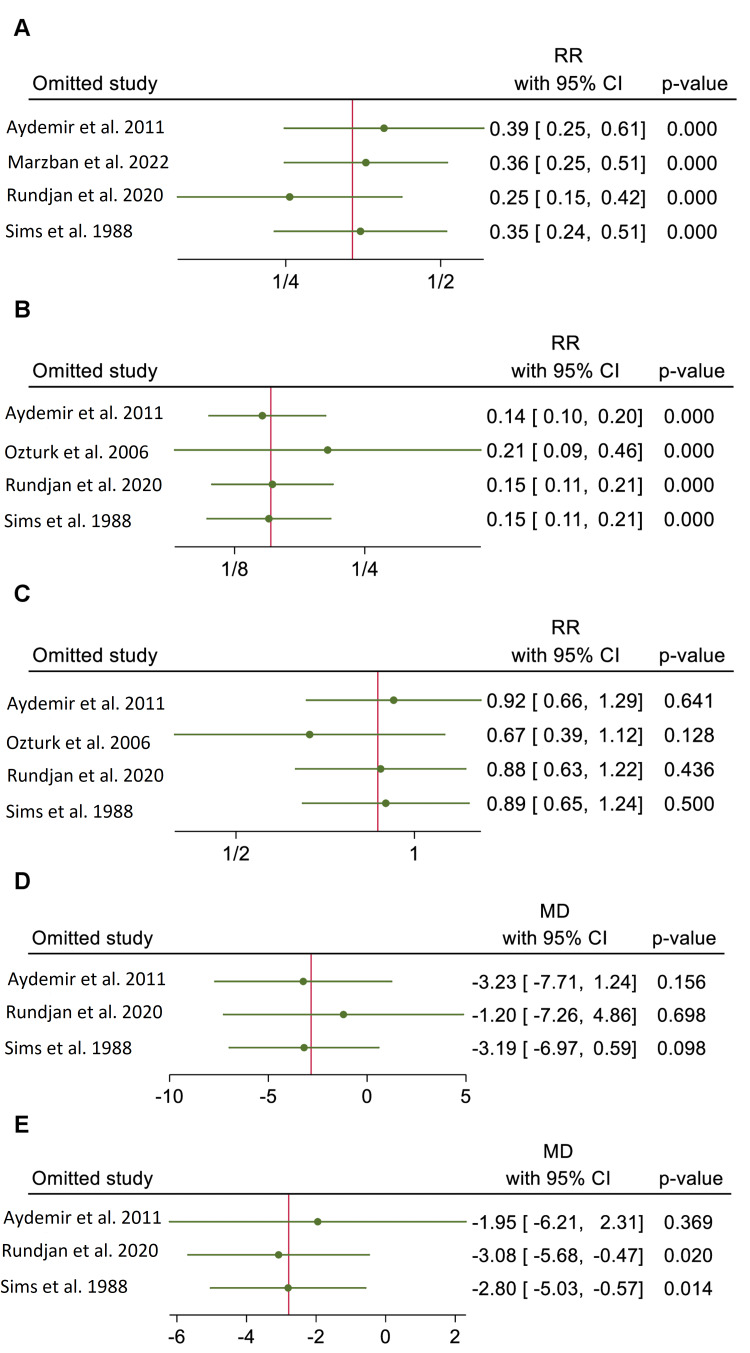
Leave-one-out sensitivity analysis of the efficacy endpoints. Charts in the image show incidence rate of fungal colonization (A), incidence rate of invasive fungal infection (B), incidence rate of mortality (C), mean length of stay in the neonatal intensive care unit (D), and mean duration of antibiotic treatment (E). RR: risk ratio; MD: mean difference

The results of the leave-one-out sensitivity analysis showed stability of all outcomes, except the outcome of the mean duration of antibiotic treatment. The omission of the study by Aydemir et al. rendered the overall effect size of the mean duration of antibiotic treatment to be statistically insignificant between both arms (n=2 RCTs, MD=-1.95 days, 95% CI {-5.03, -0.57}, p=0.369) [16]. Conversely, for all other outcomes, the omission of individual studies did not significantly impact the overall effect sizes beyond the 95% CI limits (Figure 8) [7,15-18].

Discussion

We established this systematic review and meta-analysis of RCTs with a total of 1750 patients to investigate the prophylactic efficacy of oral nystatin compared with control treatment on preventing fungal colonization and infection among VLBW infants. Our study showed significant reduction in the rates of fungal colonization and invasive fungal infection among nystatin group compared with the control group. However, the rate of mortality was not statistically different between both groups. Furthermore, the pooled analysis showed significant reduction in the duration of antibiotic administration in favor of the nystatin arm in contrast with the control arm. Nonetheless, there was no significant difference between both groups regarding reduction in the length of NICU stay.

Oral nystatin does not get absorbed and mechanistically functions by decreasing fungal colonization of the gastrointestinal tract. It has been reported to be free of major side effects in neonates. Nausea and vomiting symptoms may occasionally take place in older children when long-term and high doses are administered [19]. Despite our study findings, Kaufman argues against its use among VLBW infants, citing an absence of efficacy and safety data [20]. Ganesan et al. conducted an observational study that compared prophylactic oral nystatin with no treatment in the United Kingdom with a total sample size of 1459 preterm infants and concluded that there was a significant reduction in the rates of fungal colonization and systemic fungemia among nystatin group compared with the control group [21]. Also, Howell et al. conducted a multi-national observational study and examined the occurrence of invasive fungal infection in NICUs based on their utilization of routine prophylactic administration of nystatin [22]. The authors found that the incidence of invasive fungal infection was substantially lower in NICUs which used prophylactic nystatin contrasted with those which did not do so (0.54% versus 1.23%, respectively); the difference was statistically significant (p<0.001) [22].

Previous systematic reviews included small number of RCTs compared with our study [8]. Blyth et al. conducted a meta-analysis of RCTs that compared fluconazole and nystatin with regard to the prevention of fungal colonization and invasive fungal infection, and they found that prophylactic fluconazole and oral nystatin were both highly effective in preventing invasive fungal infection in VLBW infants [23]. Both agents were safe without significant toxicities. Also, Austin et al. concluded that the conclusion of a decrease in hazard of invasive fungal infection in VLBW infants treated with oral/topical antifungal prophylaxis should be deduced with carefulness owing to the underlying methodology-related caveats in the included studies [8]. Moreover, these reviews included only three RCTs that compared oral nystatin with control or placebo. Moreover, these reviews reported only three outcomes, namely incidence of invasive fungal colonization, mortality rate, and length of stay in NICU.

Our present research has several strengths. Most notably, this is the most comprehensive review on the topic of prophylactic nystatin administration among VLBW neonates. In comparison with a previous report, we included a total of five RCTs with a total of 1750 patients [8]. Furthermore, we have analyzed two additional important outcomes, namely incidence of fungal colonization and the duration of antibiotic administration. Overall, our results revealed that there was a significant reduction in the rate of fungal colonization and the mean duration of antibiotic administration, both of which were in favor of the nystatin arm in contrast with the control arm. Besides, all our pooled outcomes were homogeneous, reflecting no significant differences between studies. Lastly, we performed leave-one-out sensitivity analysis and showed stability of nearly all outcomes.

The present investigation has some limitations that should be acknowledged. Such limitations include the relatively small number of meta-analyzed RCTs and methodological weakness of some of the included studies. Further limitations comprise the lack of assessing publication bias. Besides, several outcomes were reported by only three studies, hence reducing the power of the pooled conclusions. Because only one RCT looked into VLBW and ELBW infants, it was not possible to perform subgroup analysis [18]. Lastly, the study protocol was not recorded in PROSPERO, hence the results could be liable to bias.

In view of the small number of meta-analyzed RCTs, future research includes the need to conduct additional, large-sized RCTs in order to strengthen the power of the pooled conclusions and solidly endorse the use of prophylactic nystatin by clinical practice guidelines. All the reviewed RCTs used the same dose of nystatin (100,000 U/mL every 8 hours) [7,15-18]. Interesting future research should examine a dose-response analysis that will identify the optimal dose that will produce the maximum and minimum efficacy and safety. Additionally, prospective research may look into for how long (duration) prophylactic administration of nystatin should be commenced. The incidence of fungal infection is largely higher in countries with low socioeconomic status [24,25]. Hence, forthcoming investigation may explore which specific cohort of VLBW infants are more likely to benefit the most from prophylactic administration of nystatin. Lastly, interesting research may investigate the efficacy of single-agent nystatin versus other active comparators (for example, fluconazole). Also, examining the combinatorial efficacy of nystatin and other active agents (for example, fluconazole) versus placebo is an exciting prospect for future research.

## Conclusions

Among VLBW infants, the use of prophylactic nystatin was significantly associated with reduction in the rate of fungal colonization, rate of invasive fungal infection, and mean duration of antibiotic administration compared with control (placebo or no treatment) group. However, there was no difference between both groups regarding the rate of mortality and mean length of NICU stay. Overall, prophylactic nystatin should therefore be used in all VLBW infants. Additional, large-sized, and high-quality RCTs are needed to validate these conclusions.
